# Canis Familiaris Papillomavirus Type 26: A Novel Papillomavirus of Dogs and the First Canine Papillomavirus within the *Omegapapillomavirus* Genus

**DOI:** 10.3390/v16040595

**Published:** 2024-04-12

**Authors:** John S. Munday, Sarah D. Bond, Susan Piripi, Susannah J. Soulsby, Matthew A. Knox

**Affiliations:** 1 Pathobiology, School of Veterinary Science, Massey University, Palmerston North 4410, New Zealand; 2 IDEXX Laboratories, Palmerston Noth 4410, New Zealand; susan-piripi@idexx.com; 3 Northland Veterinary Group, Onerahi, Whangārei 0110, New Zealand; susier@northvets.co.nz; 4 Molecular Epidemiology Laboratory, School of Veterinary Science, Massey University, Palmerston North 4410, New Zealand

**Keywords:** dog, papillomavirus, oral papilloma, CPV, canine papillomavirus, CPV26, omegapapillomavirus, evolution, Caniform

## Abstract

Domestic dogs are currently recognized as being infected by 25 different canine papillomavirus (CPV) types classified into three genera. A short sequence from a novel CPV type was amplified, along with CPV1, from a papilloma (wart) from the mouth of a dog. The entire 7499 bp genome was amplified, and CPV26 contained putative coding regions that were predicted to produce four early proteins and two late ones. The ORF L1 showed less than 62% similarity for all previously sequenced CPV types but over 69% similarity to multiple *Omegapapillomavirus* types from a variety of Caniform species including the giant panda, Weddel seal, and polar bear. Phylogenetic analysis confirmed CPV26 clusters within the *Omegapapillomavirus* genus. Specific primers were used to investigate the presence of CPV26 DNA within a series of 37 canine proliferative lesions. CPV26 DNA was amplified from one lesion, a cutaneous papilloma that also contained CPV6. This is the first time a PV type within the *Omegapapillomavirus* genus has been detected in a non-domestic species and this provides evidence that the omegapapillomaviruses infected a common ancestor of, and then co-evolved with, the Caniform species. Whether CPV26 causes disease is uncertain, but the absence of an E7 protein may suggest low pathogenicity.

## 1. Introduction

Papillomaviruses (PVs) are small, circular, double-stranded DNA viruses. As part of their normal life cycle, PVs produce proteins that promote cell growth and differentiation [1]. Most PV infections in dogs are asymptomatic [2]. However, PVs have been reported to cause both hyperplastic and, rarely, neoplastic diseases in dogs [3,4]. Hyperplastic papillomas (warts) are common in young adult dogs and most often develop in places susceptible to trauma such as the oral cavity, feet, and ears [5,6]. In papillomas, the PV infection results in marked epithelial hyperplasia resulting in folding of the epithelium and the development of either an exophytic or ‘inverted’ papilloma [7]. Current evidence suggests that there is little clinical significance to the papilloma type, and both exophytic and inverted papillomas are expected to resolve within 6 months [8]. Rarely, dogs may develop ‘persistent’ papillomas. In these cases, papillomas can become extensive and cause significant loss of function [9]. These papillomas also do not spontaneously resolve and there is currently no effective treatment. While the precise cause of persistent papillomas has not been identified, evidence from studies of humans with ‘recalcitrant warts’ suggests an underlying genetic immune deficiency that prevents an appropriate immune response against cells infected by PV [3]. While there is no clear definition of the time papillomas need to be present for to classify these as persistent, the development of additional papillomas six months after the first papillomas were observed is highly suggestive of an underlying immune dysfunction. 

Canine pigmented plaques are also caused by PV infection in dogs. Plaques are most common on the ventrum and limbs, although are less frequently confined to the head or ears [10,11]. In rare cases, plaques can coalesce and involve a large proportion of the body [12]. Immune function is considered important in disease development, and breed predispositions have been observed in pugs, miniature schnauzers, and Hungarian vizslas [13]. Early in the clinical course, plaques are sessile with the skin only mildly raised; however, the plaques become more exophytic as the lesions persist and enlarge. There are rare reports in which plaques progress to squamous cell carcinoma (SCC) in dogs [14]. 

Compared to other species, neoplasia due to PV infection appears to be rare in dogs. However, multiple oral SCCs were reported in a dog in association with canine papillomavirus (CPV) type 17 [15]. In addition, an oral papilloma caused by CPV1 was reported to progress to SCC in a dog with persistent oral papillomas [9]. Furthermore, a small proportion of viral plaques may progress to SCC, especially those caused by CPV16 [16]. 

Papillomaviruses are classified based on the sequence of the highly conserved *ORF L1*, with PVs within the same genus sharing over 60% nucleotide similarity and PVs of the same type having over 90% similarity [17]. Papillomaviruses within the same genus typically cause similar lesions within closely related species. Prior to the present study, 25 Canis familiaris papillomavirus (CPV) types had been fully sequenced and classified into one of three genera [18,19]. The *Lambdapapillomavirus* genus contains CPV1 and CPV6 and these PV types both cause papillomas [20]. CPV types within the *Taupapillomavirus* genus also cause papillomas and include CPV2, 7, and 13 [20]. The remaining fully classified CPV types are within the *Chipapillomavirus* genus. Infection by these CPV types can result in viral plaques [7]. 

Papillomaviruses typically contain five early (E) and two late (L) genes. The E1, E2, and E4 genes are all involved in efficient viral DNA replication and transcription, and are important in the initial viral replication after infection [1,21,22]. The E6 and E7 oncoproteins are most important for determining lesion development as these proteins alter the replication and differentiation of an infected cell. Depending on the PV type, these proteins prevent keratinocytes from becoming post-mitotic, promote cell division, and inhibit cell apoptosis [1,22,23]. The L1 and L2 genes encode for the capsid proteins and these genes are only expressed when the infected cell is close to the skin surface [24].

A dog developed progressive oral viral papillomas over a period of around six months. DNA from CPV1 and a novel PV type were amplified from the papillomas. Herein, the full genomic sequence of the novel PV type is described, which was classified as the first CPV type within the *Omegapapillomavirus* genus. Additional samples were evaluated and CPV26 was detected in a second papilloma, this time as a co-infection with CPV6. 

## 2. Materials and Methods

### 2.1. Initial Case Summary, Sample Collection, PCR, and DNA Sequencing

A six-month-old neutered female Huntaway dog presented to the veterinary clinic after developing oral papillomas. These were observed to grow in both size and number over the following month, at which time, azithromycin was administered. The papillomas continued to grow and became so extensive that debulking surgery was performed a month later. The papillomas rapidly recurred after surgery, requiring additional debulking surgery three months later. Samples of a wart were fixed in formalin and submitted for histology. 

DNA was extracted using a NucleoSpin DNA FFPE XS kit (Macherey-Nagel, Düren, Germany) from a sample of formalin-fixed papilloma taken from the paraffin histology block. Papillomaviral DNA was amplified using the MY09/11, FAP59/64, and the CP4/5 consensus primers as previously described [25]. DNA extracted from a canine oral squamous cell carcinoma (SCC) that contained CPV17 was used as a positive control for the reactions, while no template DNA was added to the negative controls. The amplified DNA was sequenced as previously described [26] and compared to sequences in GenBank using the BLAST tool. 

**Figure 1:**
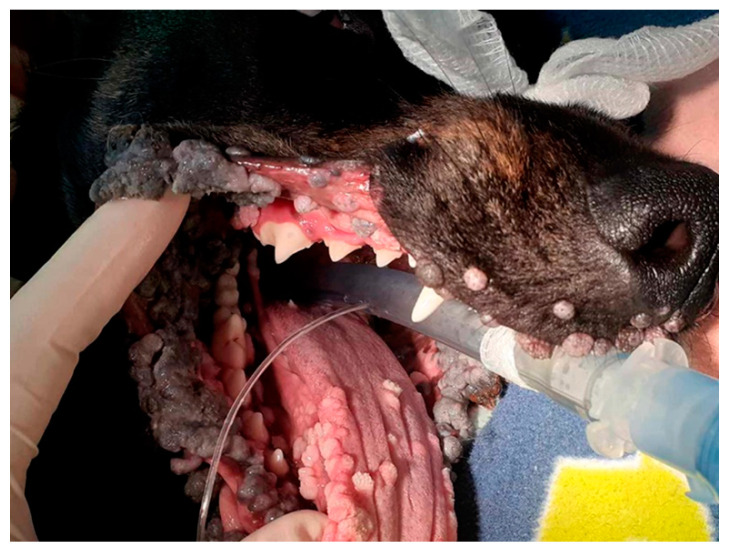
The oral papillomas were extensive and continued to develop over a 6-month period.

Due to continued proliferation of the oral papillomas, the dog underwent a further debulking surgery two months later (Figure 1). At this time, addition samples of papilloma were collected, and half were fixed in formalin for histological evaluation and the other half were unfixed. The dog continued to develop numerous large oral and lip papillomas and was euthanatized twelve months after the papillomas had been initially observed. 

### 2.2. Complete Genome Sequencing of the Novel PV

DNA was extracted from an unfixed sample of papilloma as before. To amplify the complete genome, ‘outward-facing’ primers were designed using Geneious Prime 2019.2.3 based on the short sequence of ORF L1 DNA amplified from the fixed papilloma sample. The primers (CPV26InvF 5′-CCCTTGCTGCAGCCTTAGAAG and CPV26InvR 5′-GCCTAGATTCCACCGATCCA; Integrated DNA Technologies, Coralville, IA, USA) amplified an approximately 7300 bp DNA section. Amplification was performed using repliQa HiFi ToughMix (Quantabio, Beverly, MA, USA) using an Eppendorf Mastercycler Nexus G2 (Hamburg, Germany). Initial denaturation was at 98 °C for 2 min with amplification by 40 cycles at 94 °C for 10 s, 54 °C for 15 s, and 68 °C for 8 min, followed by extension at 68 °C for 7 min. An Illumina sequencing library was prepared from the resulting PCR products using the Nextera XT DNA Library Preparation Kit (Illumina Inc., San Diego, CA, USA). Paired-end 2 × 250 bp sequencing of the DNA library was then performed on an Illumina MiSeq sequencer. Around 1.7 million reads were then assembled into a single contiguous sequence using Geneious 10.2.6 [27].

### 2.3. DNA and Protein Sequence Analysis

As the novel PV showed the greatest similarity to Ailuropoda melanoleuca papillomavirus (AmPV)1 (GenBank NC035201), the characteristics of the putative viral genes, the presence of conserved protein domains and motifs, and the presence of regulatory sequences were predicted by comparison to this PV type. This was carried out using the ‘annotate from database’ and ‘find ORF’ tools in Geneious 10.2.6 in combination with Interpro (http://www.ebi.ac.uk/interpro/, accessed on 9 January 2024). 

### 2.4. Phylogenetic Analysis

Complete genomes of 97 PV types from each of the currently recognized genera were obtained from GenBank. Nucleotide sequences for the ORF L1 were aligned using MAFFT in Geneious v10.2.6 and trimmed using ClipKIT in smart-gap mode, resulting in an alignment of 1506 nucleotides [28,29]. Maximum likelihood analysis was performed using PhyML version 3.0 [30], available on the ATGC bioinformatics platform (http://www.atgc-montpellier.fr/phyml/, accessed on 9 January 2024). Phylogenetic trees were inferred, employing subtree pruning and regrafting branch-swapping and nucleotide substitution models determined by Smart Model Selection [31]. Branch support was assessed using an approximate likelihood ratio test (aLRT) with the Shimodaira–Hasegawa-like procedure [32]. The tree was produced using a general time-reversible model with invariable sites and gamma distribution (GTR + G [0.824] + I [0.113]). Tree visualization and annotation were performed with Evolview v3 [33]. Pairwise sequence similarities were calculated from the alignment of the complete ORF L1 of the novel PV with that of other PV types.

### 2.5. Detection of CPV26 in Other Canine Papillomas and Squamous Cell Carcinomas

The pathology database at Massey University was used to identify canine cases of oral and cutaneous papillomas, and oral and cutaneous SCCs. If a dog had multiple lesions, only one lesion was used for the investigation. DNA was extracted from formalin-fixed paraffin-embedded tissue blocks as before, and the CPV4/5 primers were used to amplify PV DNA from these samples. Amplified DNA was sequenced as previously described. Specific primers to amplify a 204 bp section of CPV26 DNA were designed using Geneious Prime 2019.2.3. DNA was amplified by the primers (CPV26F 5′-GCCTAGATTCCACCGATCCA and CPV26R 5′-CACGTGGCACCAACTTTACA; Integrated DNA Technologies) using Hot FirePol^®^ Master Mix (Solis BioDyne OÜ, Tartu, Estonia) using an Eppendorf Mastercycler Nexus G2. Initial denaturation was at 95 °C for 15 min with amplification by 35 cycles at 95 °C for 20 s, 60 °C for 20 s, and 68 °C for 90 s, followed by extension at 68 °C for 90 s. DNA extracted from the original formalin-fixed sample of the oral papilloma was used as the positive control, while no template DNA was added to the negative control. 

## 3. Results

### 3.1. Histology and Initial PCR and DNA Sequencing

**Figure 2:**
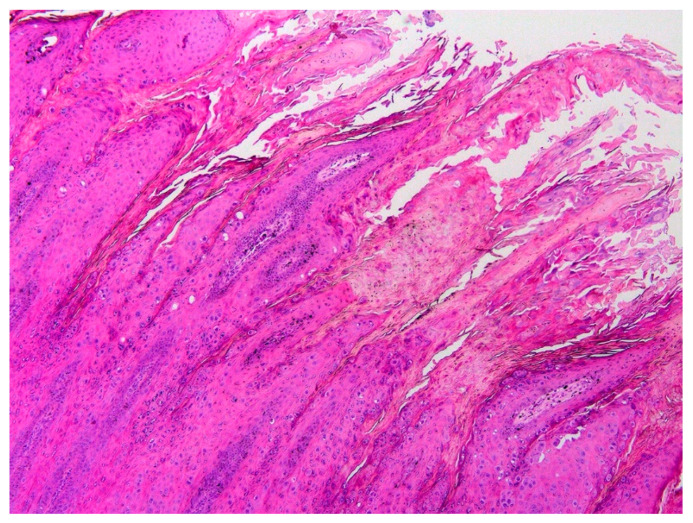
Canine oral papilloma containing both CPV1 and CPV26 DNA. Histology revealed thickened and folded epithelium covered by increased quantities of keratin. HE 25×.

**Figure 3:**
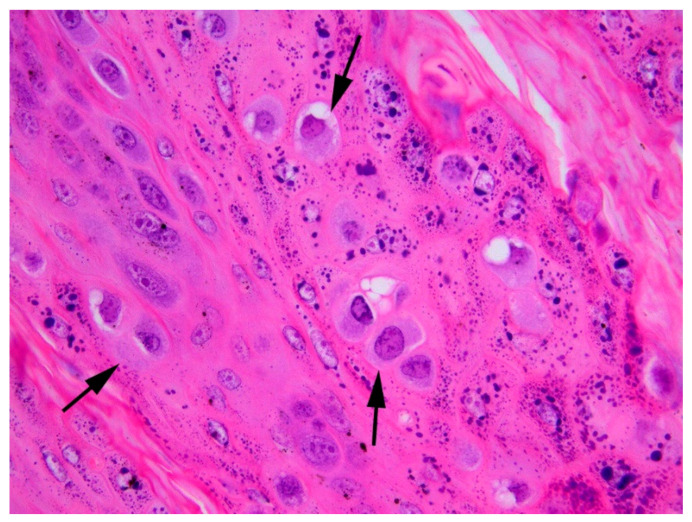
Canine oral papilloma containing both CPV1 and CPV26 DNA. The papilloma contains numerous keratinocytes that have increased quantities of blue–grey slightly granular cytoplasm (arrow). Keratohyalin granules are also prominent within the cells. HE 400×.

Histology of the papilloma revealed an approximately 1 cm diameter exophytic mass that consisted of markedly thickened folded epithelium. The epithelium was covered by increased ortho- and parakeratotic keratin, and spikes of keratin resulted in a filiform appearance to the surface of the papilloma (Figure 2). Epithelial cells close to the base of the lesions appeared mildly crowded, but no evidence of neoplastic transformation or invasion of underlying submucosa was visible. Numerous keratinocytes with cytoplasm expanded by smudged blue–grey material were visible within the thickened epithelium and keratinocytes contained prominent keratohyalin granules (Figure 3). 

Papillomaviral DNA was amplified by the FAP59/64, MY09/11, and CP4/5 primes from the papilloma and the positive control, but not the negative control reactions. The DNA amplified by the FAP59/64 and CP4/5 primers was identical to that of CPV1. However, the 450 bp section of the PV L1 ORF amplified by the MY09/11 primers was most similar to AmPV1, although the two sequences were only 70% similar. 

### 3.2. CPV26 Complete Gene Sequence

The complete genome was 7499 bp, with a GC content of 44.8%. The first nucleotide in the ORF E6 was assigned number 1 in the sequence. As this is the 26th PV type sequenced and classified from domestic dogs, it was designated CPV26. The sequence of the novel PV was deposited in GenBank under accession number PP316141.

### 3.3. Open Reading Frame Organization of CPV26 Genes

**Figure 4:**
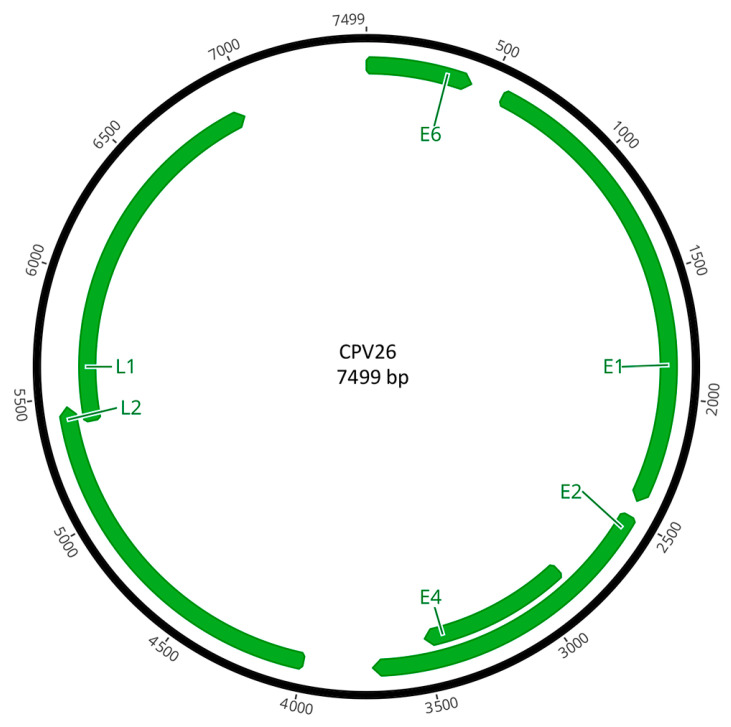
Schematic genomic organization of CPV26.

The PV was predicted to contain six ORFs that coded for four early genes (E1, E2, E4, E6) and two late genes (L1, L2; Figure 4). The predicted ORFs and characteristics of their putative protein products are shown in Table 1.

The putative E6 protein of CPV26 consists of 140 amino acids (aa) and contains two conserved zinc-binding domains (CXXC-X29-CXXC) between aa 25 and 61, and 98 and 134. The E6 sequence did not contain a PDZ-binding motif (ETQL) in its C-terminus. The predicted CPV26 E1 protein was 624 aa, with N-terminal (aa 2–119) and C-terminal (aa 329–615) ATP-dependent helicase domains. The C-terminal domain contained the conserved ATP-binding site (GPPNTGKS) for the helicase at aa 453–460. The binding of cyclin/cyclin-dependent kinase complexes to E1 is required for the initiation of PV DNA replication [34] and the predicted E1 protein had a single cyclin A interaction site (RXL) at aa 104–106. The CPV26 E2 protein was 410 aa in length, consisting of an N-terminal transactivation helicase domain (aa 1–149) and a C-terminal viral DNA binding domain (aa 333–407). Unlike some other PV types, there was no leucine-zipper domain (LX6LX6LX6L) in the putative CPV26 E2 protein [35]. The putative CPV26 ORF E4 was present within the ORF E2 region, but in a different translation frame. The presence of an ORF E4 protein has not been confirmed in other omegapapillomaviruses and a BLAST search of the amino acid sequence from the putative CPV26 ORF E4 revealed no significant similarities. The putative CPV26 E4 has a proline content of 12.1% and is also serine-rich (12.4%) with a repeat region from aa 142 to 152. A similar ‘E4-like’ region was identified previously in a different omegapapillomavirus (EF536349.1) and, like the CPV26 sequence, also contained a serine repeat region.

The late region encodes two viral capsid proteins, L1 (504 aa) and L2 (487 aa). Both proteins contain a high proportion of positively charged residues (K and R) in the C-terminal end. The highly conserved Y-R dipeptide motif was present in L1 at aa 67–70, 418–419, and 474–475. The predicted L2 protein also contained two conserved N-terminus furin cleavage motifs between aa 7 and 10, and 17 and 20, and six conserved C-terminus L1-binding sites at aa 82–85, 99–102, 169–172, 442–445, 445–448, and 448–451 (PXXP motif).

The long control region (LCR) encompasses 539 bp (nt 6962–7499) between L1 and E6. The CPV26 LCR contains two putative E2 binding sites (E2BS), with a consensus sequence ACCN6GGT, at nt 7171 and 7448, but the expected E1 binding sites were not present.

### 3.4. Phylogenetic Analysis of CPV26

**Figure 5:**
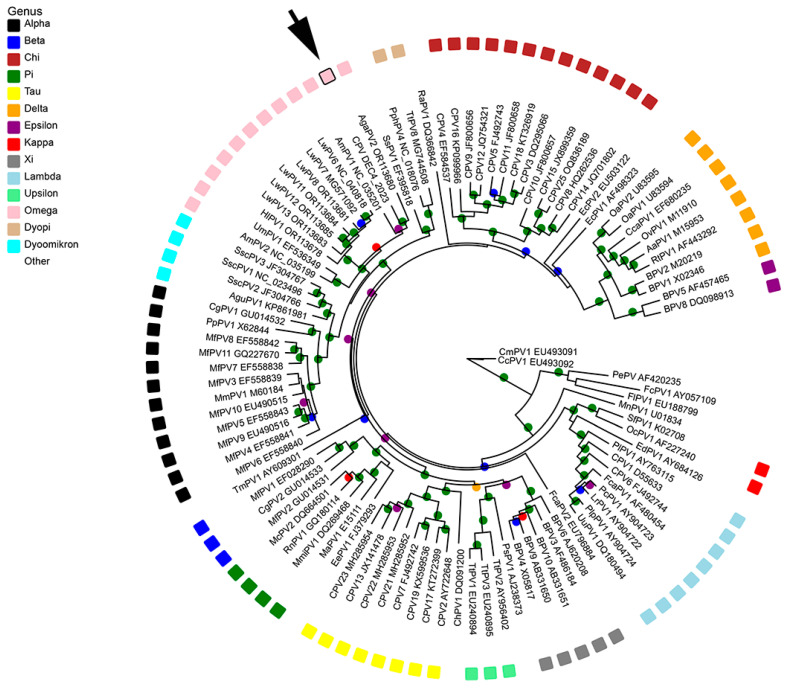
Unrooted maximum likelihood phylogeny based on concatenated nucleotide alignment of CPV26 L1 ORF sequence (arrow) with 97 other papillomavirus (PV) types of different species and genera. Accession numbers for sequences used are included. Abbreviations used include Alces alces papillomavirus, AaPV; Arctocephalus gazella papillomavirus, AgaPV; Alouatta guariba papillomavirus, AguPV; Ailuropoda melanoleuca papillomavirus, AmPV; Bovine papillomavirus, BPV; Capreolus capreolus papillomavirus, CcaPV; Caretta caretta papillomavirus, CcPV; Colobus guereza papillomavirus, CgPV; Capra hircus papillomavirus, ChPV; Chelonia mydas papillomavirus, CmPV; Canine papillomavirus, CPV; Equus caballus -+papillomavirus, EcPV; Erethizon dorsatum papillomavirus, EdPV; Erinaceus europaeus papillomavirus, EePV; Felis catus papillomavirus, FcaPV; Fringilla coelebs papillomavirus, FcPV; Francolinus leucoscepus papillomavirus, FlPV; Hydrurga leptonyx papillomavirus, HlPV; Lynx rufus papillomavirus, LrPV. Leptonychotes weddellii papillomavirus LwPV; Mesocricetus auratus papillomavirus, MaPV; Mastomys coucha papillomavirus, McPV; Macaca fascicularis papillomavirus, MfPV; Micromys minutus papillomavirus, MmiPV; Macaca mulata papillomavirus, MmPV; Multimammate rat papillomavirus, MnPV; Ovis aries papillomavirus, OaPV; Oryctolagus cuniculus papillomavirus, OcPV; Odocoileus virginianus papillomavirus, OvPV; Puma concolor papillomavirus, PcPV; Psittacus erithacus timneh papillomavirus, PePV; Procyon lotor papillomavirus, PlPV; Panthera leo persica papillomavirus, PlpPV; Phocoena phocoena papillomavirus, PphPV; Pan paniscus papillomavirus, PpPV, Phocoena spinipinnis papillomavirus, PsPV; Rousettus aegyptiacus papillomavirus, RaPV; Rattus norvegicus papillomavirus, RnPV; Rangifer tarandus papillomavirus, RtPV; Sylvilagus floridanus papillomavirus, SfPV; Saimiri sciureus papillomavirus, SscPV; Sus scrofa papillomavirus. SsPV; Tursiops truncatus papillomavirus, TtPV; Trichechus manatus latirostris papillomavirus, TmPV; Ursus maritimus papillomavirus, UmPV; and Uncia uncia papillomavirus, UuPV. Internal branches are colored based on inferred bootstrap support values, as determined using an approximate likelihood ratio test (aLRT) with the Shimodaira–Hasegawa-like procedure. Scale bar indicates genetic distance (nucleotide substitutions per site).

The maximum likelihood tree comparing CPV26 with other PV types from GenBank showed CPV26 clustered within the *Omegapapillomavirus* genus (Figure 5).

### 3.5. CPV26 Sequence Similarity to Other Papillomaviruses

The ORF L1 of the novel canine PV was most similar to that of a PV type from a giant panda (AmPV1, 72.2%; Table 2). The next most similar sequences were from PV types from Weddel seals (LwPV6, 70%); Antarctic fur seals (AgaPV2, 69.2%), polar bears (UmPV1, 69.0%), and Leopard seals (HlPV1, 66.8%). In contrast, the ORF L1 of CPV26 had <62% similarity to any of the previously reported CPV types. When the most similar CPVs in each of the three previously established CPV genera were compared, CPV26 had 61.6% similarity to CPV1 (Lambdapapillomavirus), 60.6% to CPV7 (Taupapillomavirus), and 61.6% to CPV10 (Chipapillomavirus).

### 3.6. Detection of CPV26 in Other Canine Lesions

**Figure 6:**
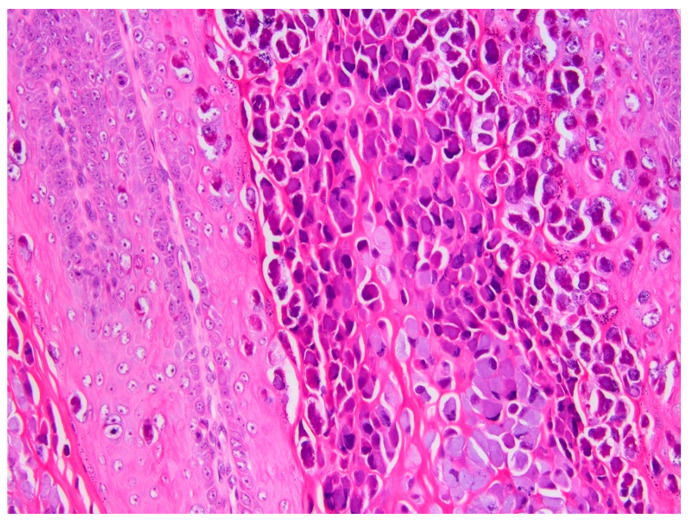
Canine cutaneous papilloma containing both CPV6 and CPV26 DNA. The papilloma contains numerous cells with blue–grey cytoplasm, but also prominent eosinophilic cytoplasmic bodies. The papilloma is consistent with a Le Net subtype. HE 200×.

A total of 37 additional hyperplastic and neoplastic lesions were evaluated. These included 22 oral lesions and 15 cutaneous lesions. The results are summarized in Table 3. CPV26 was detected in one additional lesion. This was a cutaneous papilloma that also contained CPV6 DNA. Histological evaluation of this lesion revealed typical thickening and folding of the epithelium. The papilloma contained prominent large eosinophilic cytoplasmic bodies (Figure 6) consistent with a Le Net subtype papilloma [36]. While additional studies are required, the authors have previously observed these cell changes in papillomas that contain CPV6 DNA.

## 4. Discussion

CPV26 was detected in two papillomas in this study. However, the novel PV was detected as a co-infection within both lesions. The detection of multiple PV types makes it impossible to definitively determine whether both PVs caused the papilloma, or one of the PV types caused the papilloma and the other was present as an incidental infection. The papillomas that contained CPV26 did not have any additional histological features that were not present in papillomas caused only by CPV1 or CPV6. While not conclusive, the lack of additional histological features in papillomas that contained CPV26 suggests that this PV may have been present as an incidental infection in these lesions.

The *ORF L1* of the novel PV was most similar to that of AmPV1. This papillomavirus was detected as an incidental finding in the nasopharyngeal secretions of a giant panda [37]. Other closely related PVs to CPV26 include PVs that were detected from buccal or vaginal swabs of clinically normal Antarctic fur seals, Weddel fur seals, and leopard seals [38]. However, CPV26 also has high similarity to UmPV1. This papillomavirus was detected in a raised oral lesion in a polar bear, although the lesion was not confirmed by histology to be a viral papilloma [39]. Overall, it appears most of the PV types closely related to CPV26 do not cause visible lesions. This adds to the evidence that the papillomas in the present cases may have been primarily caused by the detected *Lambdapapillomavirus* types with CPV26 having little, or no, role in lesion development.

The novel canine PV type is clustered within the *Omegapapillomavirus* genus. This genus initially only contained the polar bear PV type, UmPV1 [18]. The genus was subsequently expanded after AmPV1 was detected in a giant panda. PV types within the same genus usually have closely related hosts [17]. As polar bears are the closest extant relative to the giant panda [37], both species being infected by *Omegapapillomavirus* types would be predicted. However, the detection of omegapapillomaviruses infecting multiple seal species and now dogs is less expected. However, all species known to be infected by omegapapillomavirses are within the Caniformia suborder. As PVs are thought to be ancient viruses which co-evolved with their hosts [40], the detection of a canine *Omegapapillomavirus* type suggests these PVs may have infected a common ancestor of the Caniformia. The omegapapillomaviruses then co-evolved as these animals diversified into Canidae, Ursidae, and Pinnipedia [41]. The initial infection must have occurred at least 50 million years ago as this is when the pinnipeds are thought to have diverged from other caniformia clades [42].

Unlike other CPVs, but like other omegapapillomaviruses, CPV26 did not contain an identifiable *ORF E7* [37,39]. The most established function of the E7 protein is to promote cell division by interacting with pRb to remove an important cell checkpoint [43]. Indeed, in humans and cats, immunohistochemical detection of reduced pRb and subsequent marked increase in p16 protein are considered hallmarks of PV-induced neoplasia [44,45]. While other PV proteins can also promote cell proliferation [46], the absence of an *ORF E7* in CPV26 is also consistent with this PV type not being able to induce enough cell hyperplasia to cause visible lesions in dogs.

Whether or not the PVs within the *Omegapapillomavirus* genus express E4 protein is currently uncertain. While no *ORF E4* was identified in AmPV1 or in the PV types detected in seals, a region was identified as “similar to E4” in UmPV1 [39]. CPV26 also contained an area that appears likely to code for an E4 protein. The possible E4 proteins of both UmPV1 and CPV26 contained long serine repeat regions that have not been reported in E4 proteins from other PV genera.

In addition to the initial papilloma, a total of 37 additional samples of proliferative lesions from dogs were evaluated for the presence of CPV26. The novel PV type was detected in the initial papilloma and one other lesion, with an overall detection rate of around 5%. As there have been numerous studies investigating the presence of PV DNA within the skin of dogs, it is possibly surprising that this PV type has not been previously detected. However, in the present study, CPV26 was restricted to papillomas rather than other types of skin lesions. Therefore, it is possible that the increased keratinocyte replication within a papilloma is required before sufficient CPV26 copies are produced to enable detection by conventional PCR. Additionally, CPV26 was only detected in the initial papilloma because of the use of the MY09/11 primers as both the CPV4/5 and FAP59/64 primers amplified CPV1. This suggests the MY09/11 primers had greater affinity for CPV26 than CPV1. However, it appears likely that if only small amounts of CPV26 DNA had been present in the papilloma, the co-infection by CPV26 may have remained undetected.

The dog in the initial case developed persistent oral papillomas that eventually resulted in euthanasia. This presentation of papillomas is only rarely seen and is thought to develop because of an inherited inability to mount an appropriate immune response against PV infection [3]. The absence of a normal immune response may have allowed abnormally high replication of CPV26 within the papillomas. This abnormally high replication could also explain why CPV26 was detected using consensus primers in the present study, but in no previous investigation. While CPV26 was also detected in a papilloma from a dog with an apparently normal immune system, this was only detected using the specific PCR primers that will allow detection of a co-infection, even if the lesion contains markedly different amounts of DNA from each of the PV types.

## 5. Conclusions

This is the first report of CPV26, a novel PV type that appears to be of low pathogenicity in dogs. CPV26 is the first CPV type that is not classified within one of the three currently recognized PV genera of dogs. Instead CPV26 was classified within the *Omegapapillomavirus* genera and this PV type probably co-evolved with a common Caniformia ancestor.

## Figures and Tables

**Table 1:** Predicted ORFs in the canine papillomavirus type 26 genome; pI indicates the isoelectric point.

ORF	ORF Location	Length (nt)	Length (aa)	Molecular Mass (kDa)	pI
E1	554–2428	1875	624	70.33	4.81
E2	2493–3725	1233	410	46.15	9.08
E4	2851–3498	648	215	23.72	8.91
E6	1–423	423	140	16.61	7.66
L1	5447–6961	1515	504	57.00	7.09
L2	4003–5466	1464	487	51.21	6.85

**Table 2:** Percentage identity between the proposed CPV26 and other papillomaviruses based on the pairwise nucleotide alignments of the papillomavirus *ORF L1*. The alignments were performed in Geneious Prime 2019.2.3 using default parameters.

Papillomavirus	Host Species	Classification	L1 Similarity (%)
Ailuropoda melanoleuca papillomavirus 1 (NC_035201)	Giant Panda	Omegapapillomavirus	72.2
Leptonychotes weddellii papillomavirus 6 (NC_040818)	Weddel fur seal	Omegapapillomavirus	70.0
Arctocephalus gazella papillomavirus 2 (OR113680)	Antarctic fur seal	Omegapapillomavirus	69.2
Ursus maritimus Papillomavirus 1 (EF536349)	Polar Bear	Omegapapillomavirus	69.0
Hydrurga leptonyx papillomavirus 1 (OR113678)	Leopard seal	Omegapapillomavirus	66.8
Macaca mulata papillomavirus 1 (M60184)	Rhesus macaque	Alphapapillomavrius	63.8
Canine familiaris papillomavirus 1 (D55633)	Domestic dog	Lambdapapillomavirus	61.6
Canine familiaris papillomavirus 10 (JF800657)	Domestic dog	Chipapillomavirus	61.6
Canine familiaris papillomavirus 7 (FJ492742)	Domestic dog	Taupapillomavirus	60.6

**Table 3:** Summary of 37 canine proliferative lesions evaluated for the presence of canine papillomavirus (CPV) DNA using the CP4/5 consensus primers as well as primers specific for CPV26. SCC represents squamous cell carcinoma.

Lesion	CPV1 Only	CPV2 Only	CPV6 Only	CPV6 and 26	No CPV DNA	Total Number
Oral papilloma	1	0	0	0	0	1
Oral SCC	0	0	0	0	21	21
Cutaneous papilloma	6	1	1	1	0	9
Cutaneous SCC	2	0	0	0	4	6
All lesions	9	1	1	1	25	37

## Data Availability

The raw data supporting the conclusions of this article will be made available by the authors on request.
